# HES 130/0.4 impairs haemostasis and stimulates pro-inflammatory blood platelet function

**DOI:** 10.1186/cc8223

**Published:** 2009-12-22

**Authors:** Maik Sossdorf, Sascha Marx, Barbara Schaarschmidt, Gordon P Otto, Ralf A Claus, Konrad Reinhart, Christiane S Hartog, Wolfgang Lösche

**Affiliations:** 1 Department of Anaesthesiology and Intensive Care Therapy, Jena University Hospital, Erlanger Allee 101, D-07740 Jena, Germany

## Abstract

**Introduction:**

Hydroxyethyl starch (HES) solutions are widely used for volume replacement therapy but are also known to compromise coagulation, impair renal function and increase long-term mortality. To test the hypotheses that HES 130/0.4 has fewer adverse effects than HES 200/0.5 and exerts anti-inflammatory properties, we compared the effects of HES 130/0.4, HES 200/0.5 and saline on *in vitro *haemostasis and pro-inflammatory platelet function.

**Methods:**

Whole blood samples from healthy volunteers were mixed with 6% HES 130/0.4, 10% HES 200/0.5, or normal saline to achieve a final haemodilution rate of 10% or 40%. Haemostatic capacity was characterised by thromboelastography (ROTEM) and measurement for FXIIIa activity. Platelet activation and pro-inflammatory platelet functions were characterised by flow cytometry measuring the platelet activation marker CD62P and binding of fibrinogen to platelets as well as the formation of heterotypic platelet-leukocyte conjugates.

**Results:**

Compared with saline, HES 130/0.4 dose-dependently impaired formation and firmness of the fibrin clot but did not affect the fibrin crosslinking activity of FXIIIa. At 40% but not at 10% haemodilution rate, HES 200/0.5 also increased platelet fibrinogen binding and both HES solutions increased expression of CD62P, the main receptor for platelet-leukocyte adhesion. HES 130/0.4 but not HES 200/0.5 increased formation of platelet-neutrophil conjugates and, to a lesser degree, platelet-monocyte conjugates.

**Conclusions:**

Our data demonstrate that HES 130/0.4 has similar adverse effects as HES 200/0.5. In particular, both types of HES impair coagulation capacity and stimulate, rather than attenuate, pro-inflammatory platelet function.

## Introduction

Hydroxyethyl starch (HES) solutions are widely used for volume replacement therapy in anaesthesiology and intensive care medicine despite a lack of clinical superiority over crystalloid solutions in these patients by meta-analysis [1] or in large clinical trials [2]. HES, moreover, is associated with adverse effects such as impairment of coagulation [3], renal function [4] and long-term mortality [2]. Increased bleeding risk after infusion of HES is believed to be due not only to haemodilution but also to direct and indirect effects of HES on components of the haemostatic systems: inhibition of blood platelet function [5,6], decrease of coagulation factors such as Von Willebrand factor and factor VIII [3], decrease of plasma fibrinogen level or enhanced fibrinolysis [7-9]. However, the detailed pathomechanisms are not clear. Adverse effects of HES on haemostasis were found to depend on the *in vivo *molecular weight and the degree of hydroxylation [3,10,11]. The most modern HES with a mean molecular weight of 130 kDa and a mean degree of substitution of 0.4 (HES 130/0.4) is therefore claimed to have fewer adverse effects on haemostasis and renal function than formerly used HES solutions [12,13]. Furthermore, HES 130/0.4 has been reported to have some anti-inflammatory effects that might provide benefit to patients with systemic inflammation and sepsis [14-16]. Recent *ex vivo *studies, however, have shown that HES 130/0.4 does still impair haemostasis and platelet function and led to severe blood loss in an animal model of acute liver bleeding compared with Ringer lactate [17-19].

To test the hypotheses that HES 130/0.4 causes less coagulopathy than HES 200/0.5 and exerts some anti-inflammatory activity, we studied the effects of HES 130/0.4, HES 200/0.5 and saline on *in vitro *haemostasis by ROTEM thromboelastography (Pentapharm GmbH, Munich, Germany). Furthermore, we measured surface expression of the platelet granule membrane protein CD62P as well as the adhesion of platelets to leukocytes as markers of pro-inflammatory platelet function [20,21].

## Materials and methods

After approval by the local ethics committee and written informed consent, blood samples from 14 healthy male volunteers (22 to 61 years old) were obtained by a clean puncture of an antecubital vein and were anticoagulated by either sodium citrate (final concentration of 10.6 mM) or recombinant hirudin (50 μg/mL). Blood samples were then diluted with HES 130/0.4 (Voluven 6%; Fresenius Kabi AG, Bad Homburg, Germany), HES 200/0.5 (HAES sterile 10%; Fresenius Kabi AG) or sterile saline to obtain haemodilution rates of 10% and 40%. Thus, the final molar concentrations of HES 130/0.4 and HES 200/0.5 in the diluted blood samples were comparable. The samples were kept under gentle agitation for 15 minutes at 37°C prior to further analysis.

### ROTEM analysis and FXIII activity measurement

ROTEM was carried out according to the manufacturer's instructions in samples of citrated blood using equipment and test kits provided by Pentapharm GmbH. The following tests were performed: EXTEM^® ^to measure clot formation triggered by the activation of the extrinsic, tissue factor-dependent pathway and FIBTEM^®^, which is based on EXTEM but contains cytochalasin D to inhibit the contribution of platelets to measure the contribution of fibrin(ogen) to the clot firmness [22]. ROTEM provides a continuous measure of the clot firmness, the amplitude of which is given in millimetres. By digital data processing, the following typical variables are obtained: clotting time (CT), which is the time from start of measurement until the onset of clotting; clot formation time (CFT), which is the time between onset of clotting and the moment when clot firmness reaches an amplitude of 20 mm; the maximum clot formation rate (CFR), which is the maximum rise in clot firmness and is given as angle α; and maximum clot firmness (MCF), which corresponds to the maximum amplitude of the curve. If fibrinolysis occurs, MCF is reduced with time, and a lysis rate can be calculated as a percentage of MCF.

The activity of factor FXIIIa, which stabilises the haemostatic clot by crosslinking fibrin fibres [23,24], was determined in plasma samples obtained from blood after dilution with HES or saline. The activity was measured using a fluorogenic substrate (Fluorescent FXIII Assay; Zedira GmbH, Darmstadt, Germany). The activities are given as a percentage related to plasma samples obtained from undiluted blood.

### Flow cytometry

Flow cytometry was used to study the effects of HES on markers of platelet activation (that is, the surface expression of CD62P and the binding of fibrinogen to its activated receptor on the platelet surface [α_IIb_β_3 _integrin]) as well as the adhesion of platelets to leukocytes [25,26]. To minimise platelet-platelet aggregation, the samples were diluted 1:5 with calcium-free Hanks' balanced salt solution (HBSS). Platelet activation studies were done in hirudinised blood samples with adenosine diphosphate (ADP) (5 μM), a thrombin receptor antagonist (thrombin receptor-activating peptide, or TRAP) (25 μM) or saline as control. After the samples were incubated for 5 minutes at 37°C, aliquots were mixed with anti-CD42a-PE and one of the following fluorescence-labelled antibodies: anti-CD62P-FITC, anti-CD45-FITC (both from Becton Dickinson, Heidelberg, Germany) or anti-fibrinogen-FITC (WAK-Chemie, Bad Sodenam Taunus, Germany; all antibody dilutions were 5 μL/100 μL). All samples were incubated for 15 minutes in the dark at room temperature.

Samples labelled with CD62P-FITC antibody were mixed with 1 mL of phosphate-buffered saline (PBS) containing 1% paraformaldehyde (PFA) for cell fixation and kept on ice until flow cytometry analysis (FACScan with CellQuest Pro software; Becton Dickinson). Samples stained with fibrinogen-FITC antibody were mixed with 2.5 mL of HBSS. After centrifugation for 8 minutes at 350 *g*, the cells were resuspended in 0.5 mL of PBS/1% PFA and kept on ice until the flow cytometry measurement.

For the detection of platelet-leukocyte interactions, erythrocytes were lysed with prewarmed FACS Lysing solution (Becton Dickinson) for 5 minutes at 37°C. The reaction was stopped by the addition of 1.5 mL of HBSS. After centrifugation, the cells were resuspended in 0.5 mL of PBS/1% PFA and kept on ice until analysis.

Platelets were identified according to their forward and sideward light scatter characteristics and binding of the platelet-specific anti-CD42a and were analysed for anti-CD62P or fibrinogen binding. Neutrophils, lymphocytes and monocytes were identified by their scatter characteristics and the binding of the leukocyte-specific anti-CD45 and analysed for CD42a as a measure for the formation of platelet-leukocyte conjugates. Fluorescence-labelled isotype-matched IgG antibodies were used to correct for non-specific binding of the specific antibodies.

### Statistics

Results are given as mean ± standard error of mean. All data were examined for normal distribution using the Shapiro-Wilk test. Significances of normally distributed data were identified using analysis of variance for repeated measures followed by *post hoc *comparisons using the *t *test for paired samples. The level of significance was adjusted according to Bonferroni correction. Statistical analyses of non-normally distributed data were performed by the non-parametric Friedman test following the Wilcoxon rank sum test adjusted according to Bonferroni-Holm.

## Results

### ROTEM and FXIIIa

**Figure 1:**
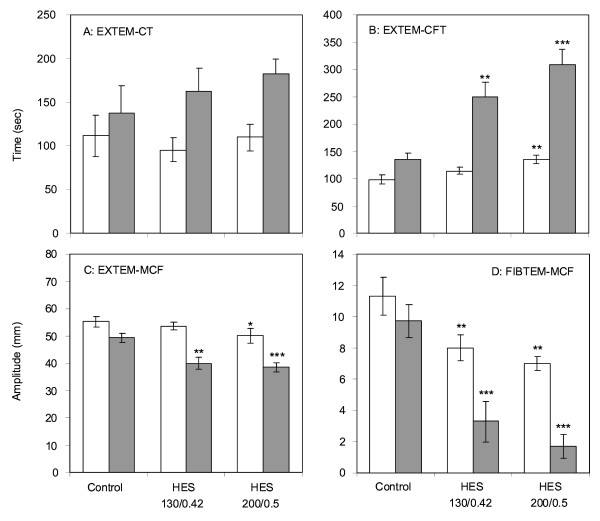
Effects of hydroxyethyl starch (HES) 130/0.4 and HES 200/0.5 on *in vitro *clot formation in comparison with saline after haemodilution rates of 10% (white) or 40% (grey) with either HES solution or saline (Control). Clotting time **(a)**, clot formation time **(b) **and maximum clot firmness (MCF) in EXTEM **(c) **as well as MCF in FIBTEM **(d) **were determined by ROTEM. Data are given as mean ± standard error of the mean obtained from six identical experiments. Significant differences between saline and HES 130/0.4 or HES 200/0.5: *P < 0.05, **P < 0.005, ***P < 0.001.

As shown in Figure 1, the effect of HES 130/0.4 and HES 200/0.5 on CT was not significantly different from that of saline (Figure 1a). With respect to the other ROTEM variables, both types of HES had significant effects compared with saline. At a haemodilution rate of 40% with HES 130/0.4, CFT was nearly doubled (Figure 1b), and MCF in EXTEM and FIBTEM were reduced by about 20% and 65%, respectively, compared with saline (Figure 1c, d). There was also a significant reduction in FIBTEM-MCF at a haemodilution rate of 10% (Figure 1d). CFR and MCF were similarly affected by HES 130/0.4, and there was no evidence of compound induced fibrinolysis (data not shown). Although we observed a trend for increased effects of HES 200/0.5, none of these effects was significantly different from those observed with HES 130/0.4 (Figure 1a-d). FXIIIa activity did not significantly differ between the blood samples diluted with saline, HES 130/0.4 or HES 200/0.5. At a 40% haemodilution rate, FXIIIa activities amounted to 60.2% ± 8.4%, 62.7% ± 13.3% and 56.6% ± 15.7%, respectively.

### Flow cytometry

**Figure 2:**
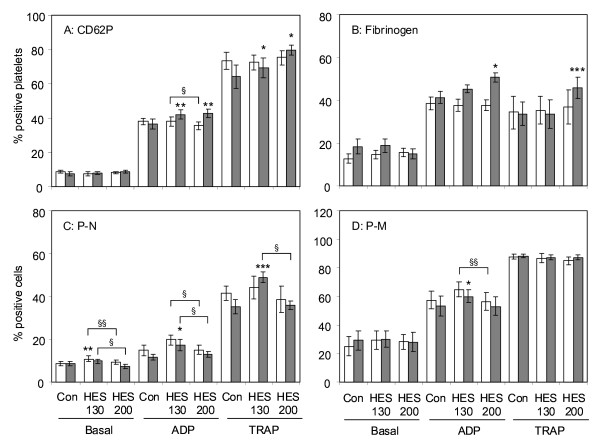
Effects of hydroxyethyl starch (HES) 130/0.4 and HES 200/0.5 on platelet activation markers and formation of platelet-leukocyte conjugates. After haemodilution of 10% (white) or 40% (grey) with either HES solution or saline (Con), platelets were activated by adenosine diphosphate (ADP) (5 μM) or thrombin receptor-activating peptide (TRAP) (25 μM) for 5 minutes or remained inactivated (Basal). CD62P expression **(a) **and fibrinogen binding to platelets **(b) **as well as platelet-neutrophil (P-N) **(c) **and platelet-monocyte (P-M) **(d) **conjugates were determined by flow cytometry. The results are expressed as the relative numbers of platelets or leukocytes that were positive for CD62P or fibrinogen or the platelet-specific antigen CD42a. Data are given as mean ± standard error of the mean obtained from eight identical experiments. Significant differences between saline and HES 130/0.4 or HES 200/0.5: *P < 0.05, ** *P *< 0.005, *** *P *< 0.001. Significant differences between HES 130/0.4 and HES 200/0.5: ^§^*P *< 0.05, ^§§^*P *< 0.01.

Both HES 130/0.4 and 200/0.5 at a haemodilution rate of 40% significantly increased CD62P expression when platelets were activated with either ADP or TRAP, but they did not change the basal expression level in non-activated platelets. At a haemodilution rate of 10%, neither HES preparation exerted a significant effect on CD62P expression (Figure 2a). When analysing the binding of fibrinogen to the platelet surface, significant enhancements that amounted to about 25% and 40% in ADP- or TRAP-activated platelets were observed by HES 200/0.5 but not by HES 130/0.5 (Figure 2b).

Figure 2c demonstrates the effect of the HES solutions on the binding of platelets to neutrophils. In contrast to its effects on the expression of CD62P and the binding of fibrinogen to platelets, HES 130/0.4 had a greater effect on the binding of platelets to neutrophils that is known to depend mainly on platelet CD62P but also on platelet α_IIb_β_3 _integrin [27]. Even without platelet activation, we observed a slight but significant increase of platelet-neutrophil conjugates in blood samples diluted with HES 130/0.4 compared with HES 200/0.5 in both 10% and 40% haemodilution rates. When platelets were activated by ADP or TRAP, HES 130/0.4 at a 40% haemodilution rate increased the number of platelet-neutrophil conjugates by a factor of about 1.5 when compared with controls diluted with saline. At haemodilution rates of both 10% and 40%, the numbers of platelet-neutrophil conjugates were significantly higher in samples treated with HES 130/0.4 when compared with those treated with HES 200/0.5 (Figure 2c).

In contrast to HES effects on platelet-neutrophil conjugates, we observed only marginal effects of HES on platelet-monocyte conjugates (Figure 2d) and no effects on platelet-lymphocyte conjugate formation (data not shown). Significant effects on platelet-monocyte conjugates were observed only with HES 130/0.4. At a 40% haemodilution rate and platelet activation by ADP, the number of conjugates was found to be above those measured in control samples, and at a 10% haemodilution rate, we found significantly more conjugates with HES 130/0.4 compared with HES 200/0.5.

## Discussion

The aim of our study was to test the hypotheses that HES 130/0.4 impairs haemostasis to a lesser degree than HES 200/0.5 and contributes to anti-inflammatory effects. Using ROTEM analysis on blood samples diluted by 10% or 40% from healthy volunteers, we observed a marked impairment of clot formation haemostasis with both HES 130/0.4 and HES 200/0.5 compared with saline. However, there were no significant differences between 6% HES 130/0.4 and 10% HES 200/0.05. The lack of difference may correspond to the similar molar concentrations of both HES solutions.

In line with our results, other investigators have found comparable effects of HES 130/0.4 and 200/0.5 on haemostasis, in particular *in vitro *clot formation as measured by ROTEM or SONOCLOT^® ^[28-31]. Jamnicki and colleagues [28] compared HES 130/0.4 or HES 200/0.5 in preoperative blood samples diluted to 30% or 60% from 80 patients scheduled for elective surgery. The two HES solutions showed comparable coagulation abnormalities. Konrad and colleagues [29] investigated 33% and 66% dilutions of whole blood from healthy volunteers with HES 70, HES 130 or HES 200 by SONOCLOT and found that all HES solutions significantly inhibited the early clotting stage compared with Ringer lactate whereas HES 130 impaired clot formation and retraction less than other HES solutions [29]. Thrombelastography in 45 patients performed after short-time infusions of HES 130, HES 200 or 4% human albumin immediately after cardiac surgery showed prolonged CFT and decreased MCF for both HES solutions whereas no changes were observed after human albumin [30]. In blood samples from volunteers diluted to 10%, 20% and 30% with normal saline, HES 130 or HES 200, both HES solutions showed noticeably inhibitory effects on platelet function whereas HES 200 had a greater effect on blood cells and plasma separation [17]. With regard to previous studies that found comparable effects of HES 130 and HES 200, none so far have compared the effects of the two HES solutions on all of the measured variables in parallel in a well-defined *in vitro *system. Furthermore, we used haemodilutions of 10% and 40% to comply more closely with conditions *in vivo*, whereas others diluted their blood samples to 55% [31] or 33% and 66% or used a different methodology [29]. In our study, neither of the two HES solutions had significant effects on CT, whereas CFT was significantly prolonged and MCF was decreased. The most pronounced effects of HES solutions were observed on MCF in FIBTEM. This test monitors the firmness of fibrin clots when the contribution of platelets, in particular their interaction with fibrin(ogen) and their contractile force, is removed. MCF dropped below the lower level of normal (<9 mm) at a haemodilution rate of 10%, which is indicative of high bleeding risk [32].

The mechanism for the inhibition of the fibrin network formation by HES is not yet clearly elucidated. So far, decreased thrombin formation, impaired interaction between thrombin and fibrinogen as well as an inhibition of FXIIIa activity have been discussed [6,33]. With respect to FXIIIa, we could not observe any effect of HES 200/0.5 or HES 130/0.4 on its activity. Some authors have reported that administration of HES resulted in an acquired fibrinogen deficiency that was more severe than expected than haemodilution alone. Consequently, fibrinogen supplementation was found to compensate compromised haemostasis [18,33,34]. It is difficult to believe that *in vitro *haemodilution with HES may result in a similar fibrinogen-consuming process. However, one can speculate that HES could directly interact with fibrinogen or fibrin and thus inhibit the interaction with thrombin or FXIIIa or both.

Very recently, a discussion has been started whether 6% HES 130/0.4 or 130/0.42 dissolved in physiologically balanced electrolyte solutions impairs haemostasis to a lesser extent than HES dissolved in saline. With respect to balanced HES solution, the supplementation of calcium ions seems to be of special importance [35-37]. However, in an *in vitro *setting, differences between the balanced solutions with and without calcium ions on ROTEM were observed only at a haemodilution rate of 50% [35,37]. At a 30% haemodilution rate, the reduction in MCF was similar to that which might be extrapolated from the EXTEM tests in the present study.

It was reported elsewhere that the *in vivo *half-life time of HES 130/0.42 is shorter than that of HES 200/0.5, and this may have an impact on their effects in clinical use [38]. However, it was not the aim of our *in vitro *study to look for degradation-dependent effects of both HES solutions. On the other hand, it seems to be questionable whether preincubation of blood samples with HES 130/0.42 or HES 200/0.5 for a different length of time could mimic the *in vivo *degradation process, including the accumulation of degradation products.

When added to blood samples, HES binds in a concentration-dependent manner to the surface of platelets, and this is believed to be responsible for the inhibition of platelet aggregation [6,39]. Boldt and colleagues [37] reported a significant inhibition of platelet aggregation in whole blood samples measured by impedance aggregometry at a 30% haemodilution rate with both unbalanced and balanced 6% HES 130/0.4 and 6% HES 130/0.42, respectively. Although using the same experimental setup to measure platelet aggregation, the same authors reported a significant inhibition of ADP- and collagen-induced platelet aggregation at 50% haemodilution, but not at 30% haemodilution, in a later communication [35].

Balanced HES 130/0.42 has been shown to increase the activation of the α_IIb_β_3 _integrin on the platelet surface [36]. This integrin is the platelet fibrinogen receptor, and its activation is an essential prerequisite for platelet aggregation [40]. The activation of the platelet fibrinogen receptor is in accordance with our present data on increased fibrinogen binding to ADP- or TRAP-activated platelets upon dilution of blood samples with unbalanced HES 130/0.4 or HES 200/0.5 (Figure 2b).

Another marker of platelet activation is CD62P, which is localised in resting platelets in intracellular granules and becomes translocated to the platelet surface upon platelet activation. CD62P is one of the molecules that mediate the interaction between platelets and leukocytes, and platelets have been shown to support inflammatory processes by CD62P-mediated interaction with leukocytes [20,21,41]. We could show that both HES 130/0.4 and HES 200/0.5 significantly increased surface expression of CD62P when platelets were activated by ADP or TRAP. The increase in platelet CD62P expression was accompanied by an increase in the formation of platelet-neutrophil conjugates, and this effect was seen not only after platelet activation but also with non-activated platelets. Interestingly, HES 130/0.4 was significantly more effective than HES 200/0.5 at increasing the number of platelet-neutrophil conjugates. Compared with the platelet-neutrophil interaction, the adhesion of platelets to monocytes was less affected by HES but again HES 130/0.4 exerted stronger effects than HES 200/0.5.

The findings of our *in vitro *study are limited because the analyses were performed in blood samples drawn from volunteers rather than from patients undergoing surgical interventions or requiring acute volume therapy. However, the observation of platelet activation for increased pro-inflammatory cell-cell interactions with neutrophils is novel.

## Conclusions

Our data obtained from *in vitro *experiments demonstrate that, with respect to the impairment of haemostasis, HES 130/0.4 does not differ from HES 200/0.5. Furthermore, our results do not support the claims that modified starch solutions may be beneficial due to anti-inflammatory effects. HES 130/0.4 may have a pro-inflammatory rather than an anti-inflammatory effect, at least at the level of neutrophil-mediated processes.

## Key messages

• In an *ex vivo *setting, solutions of hydroxyethyl starches impair coagulation and provoke platelet stimulation.

• The formation of fibrin, but not FXIIIa-mediated crosslinking, is critically involved in this process.

• Our observations suggest that there is no difference in impairment of coagulation and increase in pro-inflammatory response with respect to the extent of modification and molecular weight.

• Our results do not support the claims that modified starch solutions may be beneficial due to anti-inflammatory effects.

## Abbreviations

ADP: adenosine diphosphate; CFR: clot formation rate; CFT: clot formation time; CT: clotting time; FITC: fluorescein isothiocyanate; HBSS: Hanks' balanced salt solution; HES: hydroxyethyl starch; MCF: maximum clot firmness; PBS: phosphate-buffered saline; PFA: paraformaldehyde; ROTEM: rotations thromboelastometry; TRAP: thrombin receptor-activating peptide.

## Competing interests

KR received speakers' fees and an unrestricted grant for the VISEP (Efficacy of Volume Substitution and Insulin Therapy in Severe Sepsis) study from B. Braun Melsungen AG (Melsungen, Germany). All other authors declare that they have no competing interests relevant to this manuscript.

## Authors' contributions

MS and WL had the original idea for the study, were responsible for experimental analysis and data interpretation, performed statistical analyses, interpreted the results and wrote the manuscript. SM and GPO performed flow cytometric analyses of data and were involved in their interpretation. BS performed ROTEM analysis and FXIIIa analysis and was involved in their interpretation. KR, CSH and RAC provided substantial and helpful comments throughout the study, including the interpretation of results and the preparation of the manuscript. All authors read and approved the final manuscript.
